# microRNA-214 functions as a tumor suppressor in human colon cancer via the suppression of ADP-ribosylation factor-like protein 2

**DOI:** 10.3892/ol.2014.2746

**Published:** 2014-11-28

**Authors:** LI-MIN LONG, BEN-FU HE, GUO-QING HUANG, YONG-HONG GUO, YOU-SHUO LIU, JI-RONG HUO

**Affiliations:** 1 Department of Geriatrics, The Second Xiangya Hospital, Central South University, Changsha, Hunan 410011, P.R. China; 2 Department of Oncology, 421 Hospital of the People’s Liberation Army, Guangzhou, Guangdong 510318, P.R. China; 3 Department of Emergency, Xiangya Hospital, Central South University, Changsha, Hunan 410008, P.R. China; 4 Department of Gastroenterology, The Second Xiangya Hospital, Central South University, Changsha, Hunan 410011, P.R. China

**Keywords:** ADP-ribosylation factor-like protein 2, apoptosis, proliferation, miR-214, human colon cancer

## Abstract

microRNAs (miRNAs/miRs) are a conserved class of endogenous, short non-coding RNAs that post-transcriptionally regulate the expression of genes involved in diverse cellular processes. miR-214 has been reported to be associated with several cancers, including human colon cancer. However, the function of miR-214 in colon cancer development is poorly understood. In the current study, miR-214 was demonstrated to be downregulated in colon cancer tissues compared with healthy colon tissues. Functional studies showed that miR-214 overexpression results in the inhibition of cell viability, colony formation and proliferation, and the induction of cell apoptosis. ADP-ribosylation factor-like protein 2 (ARL2) is predicted to be a target candidate of miR-214. A luciferase reporter assay, western blot analysis and quantitative polymerase chain reaction were performed, which revealed that miR-214 negatively regulates ARL2 expression by targeting its 3′ untranslated region directly. In conclusion, the results of the present study revealed that miR-214 suppresses colon cancer cell growth via the suppression of ARL2, and indicated that miR-214 may present a significant potential therapeutic target for colon cancer.

## Introduction

microRNAs (miRNA/miRs) are a conserved family of small non-coding RNA molecules that are recognized as key regulators of gene expression at the post-transcriptional level via base pair binding to the binding sites of the 3′ untranslated region (UTR) of target genes, leading to target gene mRNA cleavage or translational repression (1,2). Previous studies have shown that miRNAs are involved in diverse cellular processes, including cell growth, development, apoptosis and cancer (3). The observation that ~50% of miRNAs are located in tumor-associated or fragile regions validates the hypothesis that abnormal miRNA expression is closely associated with cancer initiation and progression (4). A previous study also showed that ~60% of protein-coding genes are regulated by miRNAs (5). Depending on the potential functions of their targets in tumors, miRNAs may function as oncogenes or tumor suppressors. For example, miR-125b inhibits liver cancer cell growth and metastasis by targeting LIN28B, functioning as a tumor suppressor (6). These results indicate that miRNAs are crucial in cancer processes and may present novel biomarkers for cancer diagnosis and progression.

Human colon cancer is one of the most common malignancies and is the third leading cause of cancer-related mortality worldwide (7). However, the molecular mechanism underlying colon cancer growth and progression remains unclear. Therefore, the identification of novel molecules responsible for colon cancer development is crucial. Recent studies have demonstrated that the abnormal expression of certain miRNAs, including miR-145 (8), miR-203 (9) and miR-365 (10), is involved in colon cancer. However, the function of miR-214 in colon cancer has not yet been identified.

## Materials and methods

### Tissue samples, cell culture and transfection

A total of 24 human colon cancer tissues and paired adjacent normal tissues were obtained from the Second Xiangya Hospital (Changsha, China). Written informed consent was obtained from all colon cancer patients who were diagnosed by immunohistochemical staining and pathological diagnosis. The tissues were stored at −80°C.

The human colon cancer SW480 cells were cultured in Dulbecco’s modified Eagle’s medium (Invitrogen Life Technologies, Carlsbad, CA, USA) supplemented with 10% fetal bovine serum (FBS) and 2 mM L-glutamine (Invitrogen). SW620 cells were cultured in L-15 medium supplemented with 10% FBS. All the cells were maintained in a humidified incubator with 5% CO_2_ at 37°C. miR-214 mimics and controls were purchased from Shanghai GenePharma Co. Ltd (Shanghai, China). The cells were transfected with Lipofectamine 2000 (Invitrogen) according to the manufacturer’s instructions. This study was approved by the ethics committee of the Second Xiangya Hospital.

### RNA isolation and quantitative polymerase chain reaction (qPCR)

Total RNA was isolated using TRIzol reagent (Invitrogen) according to the manufacturer’s instructions. Next, 500 ng of RNA was used for the reverse transcription (RT) reaction and specific RT primers were used for cDNA synthesis of miR-214. U6 small nuclear B non-coding RNA (Shanghai GenePharma Co. Ltd) was used as an internal control for the normalization of miR-214. For cDNA synthesis of large oligonucleotides, oligo(dT) was used as a common primer. GAPDH (Shanghai GenePharma Co. Ltd) was used as an internal control for the normalization of ADP-ribosylation factor-like protein 2 (ARL2) expression (Shanghai GenePharma Co. Ltd). qPCR was performed using the SYBR Green PCR Master Mix (Applied Biosystems, Carlsbad, CA, USA) according to the following conditions: 95°C for 5 min, followed by 40 cycles of amplification at 95°C for 30 sec, 57°C for 30 sec and 72°C for 30 sec.

### Western blot analysis

The transfected cells were collected at 48 h post-transfection and lysed using radioimmunoprecipitation assay (RIPA) buffer [50 mM Tris-HCl (pH 8.8), 150 mM NaCl, 1% NP-40, 1% sodium deoxycholate and 0.1% SDS] for 30 min at 4°C. The protein concentration was measured using the bicinchoninic acid method. A total of 50 μg of protein was used for the analysis of ARL2 expression and GAPDH was used as a loading control. Rabbit monoclonal anti-ARL2 (1:200) and anti-GAPDH (1;1,000) (Abcam, Cambridge, MA, USA) were used as the primary antibodies. Goat anti-rabbit immunoglobulin G conjugated to horseradish peroxidase (1:1,000) was used as the secondary antibody (Abcam). The bound antibodies were detected using the Electrochemiluminescence Plus Western Blotting Detection System (GE Healthcare Bio-Sciences, Pittsburgh, PA, USA) and the chemiluminiscent signals were detected using high-performance chemiluminescence film (GE Healthcare Bio-Sciences).

### WST-1 assay

The transfected cells were plated at a density of 4×10^3^ cells/well into 96-well plates. Following transfection for 12, 24 and 48 h, the cells were incubated with WST-1 reagent (Beijing Dingguo Biotechnology Co., Ltd., Beijing, China), which is similar to 3-(4,5-dimethylthiazol-2-yl)-2,5-diphenyltetrazolium bromide, for ~1 h at 37°C. The absorbance at a wavelength of 490 nm was measured using a spectrophometer (F-4500, Hitachi, Tokyo, Japan).

### Colony formation assay

The transfected cells were seeded at a density of 200 cells/well into 12-well plates. The medium was replaced every three days until the majority of the colonies consisted of >50 cells. The colonies were then washed, fixed and stained using crystal violet (Sigma-Aldrich, St. Louis, MO, USA). Finally, images of the stained colonies were captured and the colonies were counted (G16, Canon Inc., Tokyo, Japan).

### Annexin V fluorescein isothiocyanate (FITC)/propidium iodide (PI) apoptosis assay

Camptothecin (Sigma-Aldrich) was added to the medium of the transfected cells for the induction of cell apoptosis. At 24 h post-incubation, the cells were collected and detected by an Annexin V-FITC/PI double staining kit using the BD FACSCalibur system (Becton Dickinson, Franklin Lakes, NJ, USA) according to the manufacturer’s instructions, as described previously (11).

### Cell cycle analysis

The cells were starved for 24 h post-transfection and then incubated with normal medium for an additional 24 h. Next, the cells were washed with cold phosphate-buffered saline (PBS), digested to form single cells and fixed with 70% ethanol for ≥1 h. The cells were then washed again and stained with PI (Sigma-Aldrich) supplemented with RNase A and Triton X-100 for 40 min at 37°C. Finally, the stained cells were washed and resuspended in PBS for cell cycle analysis using the BD FACSCalibur system (Becton Dickinson).

### Luciferase reporter assay

The 3′UTR of ARL2 was amplified and inserted downstream of the luciferase reporter gene. The mutant 3′UTR of ARL2 (GCUG to AAGC) was amplified using wild-type ARL2 3′UTR as the template. The cells were co-transfected with miRNA mimics and wild-type or mutant ARL2 3′UTR. Following transfection for 48 h, the cells were collected and lysed using RIPA buffer. The luciferase intensity was measured using the Dual Luciferase Reporter Assay System (Promega Corporation, Madison, WI, USA) according to the manufacturer’s instructions.

### Statistical analysis

All data are presented as the mean ± standard deviation and represent three independent experiments. The difference between groups was analyzed using the paired Student’s t-test and P<0.05 was considered to indicate a statistically significant difference.

## Results

### miR-214 downregulation in human colon cancer

**Figure 1:**
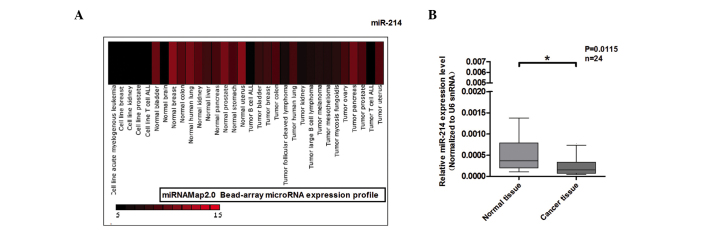
miR-214 downregulation in human colon cancer tissues. (A) The expression of miR-214 was analyzed using miRNAMap-2.0 among diverse normal tissues and cancer tissues, including colon cancer tissues. The color shades represent the relative expression levels of miR-214. (B) miR-214 expression was quantified by qPCR in 24 paired colon cancer tissues and normal tissues. U6 was used as an internal control. ^*^P<0.05. snRNA, small nuclear RNA; miRNA/miR, microRNA; qPCR, quantitative polymerase chain reaction.

To investigate the function of miR-214 in human colon cancer development, miRNAMap2.0 (12) was used for the analysis of miR-214 in diverse normal tissues and tumor tissues, including colon cancer tissues. As shown in Fig. 1A, miR-214 was found to be downregulated in colon cancer. Based on the analysis of miRNAmap2.0, qPCR was performed to detect miR-214 expression in 24 paired normal and colon cancer tissues (Fig. 1B). miR-214 expression was found to be downregulated in colon cancer. These results indicate that the abnormal expression of miR-214 may be significant in colon cancer.

### miR-214 overexpression inhibits colon cancer cell viability and colony formation

**Figure 2:**
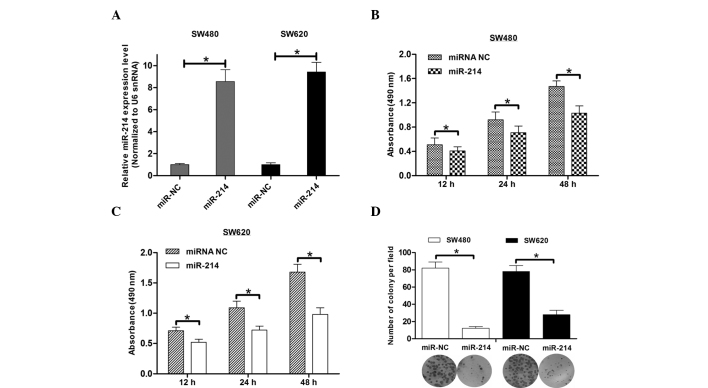
miR-214 inhibits the growth of colon cancer cells. Cells were transfected with miR-214 mimics or mimic controls and (A) qPCR was performed to investigate the overexpression of miR-214. (B–C) Cells transfected with miR-214 mimics or controls were subjected to WST-1 assay for the analysis of cell viability at various time-points. (D) Transfected cells were subjected to colony formation assay for the analysis of cell growth. The images below the bar graph show the stained colonies. ^*^P<0.05. miRNA/miR, microRNA; qPCR, quantitative polymerase chain reaction; NC, negative control; snRNA, small nuclear RNA.

To investigate the functional role of miR-214 downregulation in colon cancer, cell viability and colony formation assays were performed to analyze cell growth. The overexpression of miR-214 in the SW480 and SW620 colon cancer cells treated with miR-214 mimics was confirmed (Fig. 2A). The results from the WST-1 assay showed that miR-214 led to the inhibition of SW480 cell viability by 20–30% at various time-points compared with the miR-214 control cells (Fig. 2B). Accordingly, miR-214 inhibited the cell viability of the SW620 cells (Fig. 2C). Consistent with the effect of miR-214 on cell viability, miR-214 inhibited the number of SW480 and SW620 cell colonies by ~75 and 60%, respectively (Fig. 2D). These results indicate that miR-214 may exhibit a key function in colon cancer growth.

### miR-214 overexpression promotes colon cancer cell apoptosis

**Figure 3:**
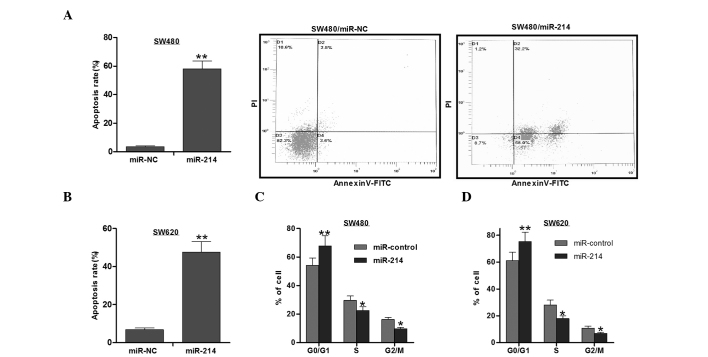
miR-214 promotes apoptosis and inhibits the proliferation of colon cancer cells. (A) An analysis of the apoptotic rate was performed using the Annexin V-FITC/PI double staining method. The apoptotic rate of the SW480 cells with miR-214 mimics was 58.0%, while that of the mimic controls was 3.6%. (B) Apoptotic rate of the SW620 cells. (C–D) The number of the transfected cells in the G_0_/G_1_, S and G_2_/M phases was counted using PI staining. ^*^P<0.05 vs. control group, ^**^P<0.01 vs. control group. FITC, fluorescein isothiocyanate; PI, propidium iodide; miRNA/miR, microRNA; NC, negative control.

The analysis of the apoptotic rate of the colon cancer cells was performed using the Annexin V-FITC/PI double staining method. As shown in Fig. 3A and B, the cells treated with miR-214 mimics exhibited a higher apoptotic rate than the cells with the miR-214 control. These results indicate that miR-214 inhibits cell growth partly through promoting cell apoptosis.

### miR-214 overexpression inhibits colon cancer cell proliferation

Cell proliferation was analyzed by cell cycle analysis using PI. It was found that miR-214 increased the number of cells in the G_1_ phase, while reducing the number of cells in the S phase, leading to G_1_/S arrest in the SW480 and SW620 cells (Fig. 3D). Therefore, the inhibition of cell proliferation may be responsible for the inhibitory role of miRNA-214 in cell growth.

### ARL2 is a direct target gene of miR-214 in colon cancer

**Figure 4:**
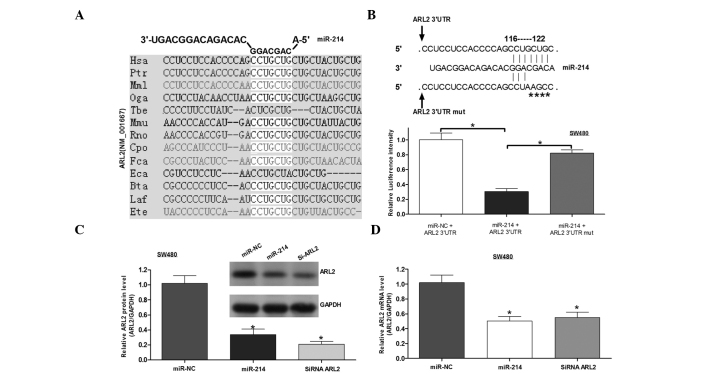
ARL2 is a direct target gene of miR-214. (A) Sequence alignment of ARL2 3′UTR potential binding sites and miR-214 seed sequence. The binding sites were identified to be conserved among species. (B) A point mutation was generated within the binding sites. The black vertical lines present the base pairing and (*) presents the mutated base. The cells were co-transfected with miR-214 mimics and wild-type or mutant ARL2 3′UTR, and the luciferase intensity was examined by using the luciferase reporter assay. (C) Western blot analysis revealed that miR-214 inhibited the ARL2 protein levels. GAPDH was used as a loading control, and the effect of ARL2 siRNA on the ARL2 protein levels was used as a positive control. (D) qPCR showed that miR-214 inhibited the ARL2 mRNA levels. GAPDH was used as an internal control, and the effect of ARL2 siRNA on the ARL2 mRNA levels was used as a positive control. ^*^P<0.05. ARL2, ADP-ribosylation factor-like protein 2; UTR, untranslated region; siRNA, small interfering RNA; qPCR, quantitative polymerase chain reaction; miRNA/miR, microRNA; NC, negative control.

To analyze the molecular mechanisms underlying the regulation of miR-214 in colon cancer growth, Targetscan and Pictar software was used to predict the target of miR-214. From the candidates, ARL2 was selected for further study. A binding site for miR-214 was identified in the 3′UTR of ARL2, and the binding sites were conserved among species (Fig. 4A). To validate whether ARL2 is a direct target of miR-214, a point mutation was generated with binding sites and cloned into the downstream region of the luciferase reporter gene. The cells were then co-transfected with miR-214 mimics and wild-type or mutant ARL2 3′UTRs. The results from the luciferase reporter assay indicated that miR-214 lead to the inhibition of the luciferase intensity of ARL2 3′UTR, whereby this inhibition was eliminated in the mutant ARL2 3′UTR (Fig. 4B). To investigate the function of miR-214 in ARL2 expression, western blot analysis and qPCR assays were performed. It was identified that miR-214 inhibited the expression of ARL2 protein and mRNA (Fig. 4C and D), similar to the function of ARL2 siRNA. These results indicated that miR-214 negatively regulates ARL2 expression by directly binding to its 3′UTR.

## Discussion

miRNAs are considered to be key regulators of protein-coding gene expression, exhibiting a crucial function in cellular processes (1). Accumulating evidence shows that the dysregulation of miRNAs is associated with cancer initiation and development (3). A previous study showed that miR-214 expression is decreased in human cervical cancer and that it inhibits cell proliferation, migration and invasion (13). In addition, miR-214 is downregulated in hepatoma and inhibits tumor angiogenesis by inducing hepatoma-derived growth factor (14). In agreement with these results, the present study analyzed the expression of miR-214 in various normal and cancer tissues, including colon cancer tissues, using miRNAmap2.0. The analysis showed that miR-214 expression was downregulated in colon cancer. In addition, miR-124 overexpression was shown to inhibit cell growth and promote cell apoptosis, functioning as a tumor suppressor. Controversy remains with regard to the function of miR-214 in tumor progression. In certain cancers, miR-214 has been shown to function as a tumor suppressor (13,14), consistent with the results of the present study. However, miR-214 has also been shown to be downregulated in ovarian cancer and to induce cell survival and cisplatin resistance by targeting the phosphate and tensin homolog 3′UTR (15), indicating that it may function as an oncogene. In addition, miR-214 has been found to be upregulated in gastric carcinoma (16), melanoma (17,18) and hepatocellular carcinoma (19), playing an important role in the promotion of tumor malignancy. These results indicate that the different roles of miR-214 may be tissue or cell-specific, and the main target genes of miR-214 may be responsible for its diverse functions.

ARL2 is a GTPase belonging to the ADP-ribosylation factor family (20) and is located on chromosome 11 (11q13). ARL2 is considered to be involved in the regulation of tubulin peptide folding and microtubule dynamics in breast cancer cells (21). Previous studies have revealed that ARL2-knockdown results in G_1_/S phase arrest and the inhibition of cell proliferation, which is a direct target of miR-16 (22). In addition, ARL2 has been reported to form a complex with the tumor suppressor, protein phosphatase 2A (PP2A), leading to changes in the phosphorylation status or the cellular sublocalization of p53, a specific PP2A target. The altered localization of p53 induces cell sensitivity to anticancer compounds in breast cancer cells (23). These results demonstrate that ARL2 exhibits an oncogenic role in disease. Based on the results reported in previous studies and the binding sites of miR-214 in the ARL2 3′UTR, in the current study, ARL2 was selected as a candidate target of miR-214. The luciferase assay indicated that miR-214 reduced the luciferase intensity controlled by ARL2 3′UTR. However, the inhibitory function of miR-214 in the mutant 3′UTR of ARL2 was eliminated. In addition, miR-214 inhibited the ARL2 protein and mRNA levels. The function of ARL2 in cell proliferation, as shown in previous studies, indicated that ARL2-knockdown may provide a phenocopy of the effect of miR-214 on colon cancer cells. These results confirm that ARL2 is a direct target gene of miR-214 and that miR-214 negatively regulates its expression.

During the investigation of the function of miR-214 in ARL2 protein and mRNA expression, the siRNA of ARL2 was used as a positive control. miR-214 was found to inhibit ARL2 expression by ~60%, similar to the inhibitory effect of siRNA on ARL2 expression (Fig. 3C and D). These results further indicated that miRNAs and siRNAs exhibit similar functions in target gene expression (24).

In conclusion, we hypothesize the following explanation for the regulation mechanism of miR-124 in colon cancer: miR-214 suppresses cell growth and promotes cell apoptosis by targeting ARL2. However, additional confirmed target genes of miR-214 may also mediate its function in colon cancer, although this requires further investigation. The results of the present study indicate that miR-214 may present a novel biomarker for the evaluation of colon cancer progression, and may present a potential miRNA-based therapeutic target for colon cancer patients.
